# Use of Essential Oils in the Diet of Lactating Cows Enhances Productivity and Reduces Methane in Free-Grazing Commercial Dairy Farms

**DOI:** 10.3390/ani15243549

**Published:** 2025-12-10

**Authors:** Juan Ignacio Oyarzún Burgos, Moira Paz Wilhelm Saldivia, Lorena Ibáñez San Martin, Ambar Madeleyn Cárdenas Vera, Roberto Bergmann Poblete, Lisseth Valeska Aravena Cofre, Benjamín Glasner Vivanco, Viviana Bustos Salgado

**Affiliations:** 1 Programa de Investigación Cero Huella, Vicerrectoria de Investigación y Post Grado, Universidad de Los Lagos, Avda. Fuchslocher 1305, Osorno 5290000, Los Lagos, Chilemoirapw@gmail.com (M.P.W.S.); cardenasv.ambar@gmail.com (A.M.C.V.); robertoignacio.bergmann@alumnos.ulagos.cl (R.B.P.); lisseth.aravena@ulagos.cl (L.V.A.C.); benjamin.glasner@ulagos.cl (B.G.V.); 2 Departamento de Acuicultura y Recursos Agroalimentarios, Universidad de Los Lagos, Avda. Fuchslocher 1305, Osorno 5290000, Los Lagos, Chile; 3 IANSA S.A., Francisco Bilbao 1860, Osorno 5310644, Los Lagos, Chile

**Keywords:** feed additives, enteric methane, grass feed, commercial farm

## Abstract

Several solutions are being explored to reduce methane intensity in dairy farms, but there is no consensus for commercial pastoral dairy systems in temperate zones. We evaluated the effects of essential oils (EO) supplementation on CH_4_ intensity and performance in dairy cows within a commercial pasture-based system in southern Chile. Thirty multiparous cows were randomly assigned to a control group and a treated group, with a general average yield of 22.3 ± 5.37 kg/d and an average parity of 3.42 ± 1.13. The treated group received concentrate supplemented with a mixture of EOs. Enteric CH_4_ emissions were measured using GreenFeed^®^. Milk yield (kg/d), composition (% fat, % protein, urea, somatic cells), plasma biochemistry, and grassland proximal analysis (NIRs) were also evaluated. Results showed a significant increase in fat-corrected milk production (4.6 kg) in the treated group during the first trial period where the grassland was highly nutritious, offering 19.8% crude protein as well as a pool of long-chain fatty acids. Additionally, CH_4_ intensity was significantly lower in the treated group (1.3 gCH_4_/ECM) during the first phase. EO supplementation strategies represent a suitable non-invasive intervention suitable for commercial grassland-based systems in southern Chile that is strongly influenced by pasture quality.

## Simple Summary

This study demonstrates, for the first time, the impact of essential oils on fat-corrected milk production and methane emissions in grassland-based cows at a commercial dairy farm in southern Chile. Thirty cows were divided into two groups, one with the herd’s usual diet and the other with the same diet but with the addition of essential oils. We measured methane, milk produced, biochemical profile, and nutritional analysis of the pastures. The cows that received the essential oil produced more milk with a higher fat content during the first phase of the trial. They also released less methane per litre of milk produced, only when the grassland had more than 19.8% protein and provided long-chain fatty acids. The findings provide strong evidence of the relationship between the nutritional quality of pasture and the productive environmental effects of these additives.

## 1. Introduction

Dairy production is projected to grow steadily at 1.7% per year over the next decade, reaching 981 million tonnes by 2028 [1]. Furthermore, food security is one of the highest priority issues in the development of Latin American countries, and dairy farming will play a key role in the countries of the Southern Hemisphere [1,2]. Livestock production places substantial pressure on the planet’s natural resources, utilizing approximately 30–60% of the global ice-free terrestrial surface and constituting a critical component in efforts toward sustainable agricultural development. The livestock sector is responsible for an estimated 14.5% of global greenhouse gas (GHG) emissions, with cattle representing the dominant source. This contribution amounts to roughly 3.8 gigatons of CO_2_ equivalent per year, primarily attributed to enteric methane emissions [3]. In temperate regions, dairy cow feeding has traditionally relied on pasture, especially where pasture growth can be sustained throughout the year, such as Ireland, New Zealand, parts of Australia, the United States, Europe, and South America [1,2,3,4]. While intensive TMR (Total Mixed Ration)-based feeding systems provide greater dietary control and can improve production, significant evidence supports pasture-based dairy production systems, often called “grass-fed” systems [2,4,5,6]. These systems offer a different nutritional profile, providing benefits over conventional milk produced through TMR methods [5].

Milk and dairy products are important food sources that provide essential nutrients [7,8], including conjugated linoleic acid, oleic acid, n-3 fatty acids, short- and medium-chain fatty acids, vitamins, and other bioactive compounds [9,10]. These nutrients can benefit human health, not only for vulnerable age groups such as children and the elderly but also for individuals with pre-existing medical conditions. Additionally, consumers generally perceive grass-fed milk as more environmentally sustainable and better for animal welfare [2]. Those remarks highlight the potential for capitalizing on dairy products produced in pastoral systems. A diverse group of consumers is willing to pay a premium for the unique qualities offered by these pastoral systems [2,5], even more so than for organic products, which are already priced high. However, these systems will require exhaustive management of the seasonality of the nutritional quality of the grassland to achieve their intended purpose as sustainable, low greenhouse gas (GHG) emitting systems [11,12]. Research has focused on reducing a specific GHG, enteric methane (CH_4_) emissions, for almost five decades through several strategies aimed to reducing emissions by modifying microbial fermentation processes within rumen [13,14]. Among the strategies for the development of sustainable livestock systems with low GHG emissions, natural additives such as essential oils have shown mixed results [15,16,17].

A common approach to essential oils inclusion has been carried out under in vitro conditions, which fail to capture the complex dynamics of the rumen in vivo and therefore do not provide reliable evidence regarding their impact on milk production [15,17,18]. Other approaches rely on experimental confined dairy systems, which also do not accurately reflect the variability in feeding practices, management conditions, or animal stress levels [17,19]. Moreover, to our knowledge, only one study conducted on a commercial dairy farm has been published to date, reporting no significant effect of EO supplementation on methane emissions [20]. On the other hand, grazing-based dairy production systems show that animal response is highly variable and significantly affected by genetic background, environment, management, and social and individual factors in contrast to what is described for confined systems [21,22,23,24]. Therefore, quantifying and mitigating enteric CH_4_ emissions from grassland-based systems presents unique challenges related to floristic composition and phenological changes throughout the year [21,22]. In addition, seasonal nutritional quality changes in grassland are becoming increasingly relevant in the context of climate change, particularly in temperate climates [25].

Southern Chile’s climatic and soil conditions favour forage growth for most of the year, offering a notable cost advantage for dairy cattle production systems [26]. However, forage growth is highly seasonal, with limited development during the winter due to low temperatures and reduced solar radiation [12,22]. It peaks in late spring and early summer, while late summer and autumn see constrained growth as temperatures and sunlight decrease [12]. Studies performed with supplemented EO showed an increase in productivity [27,28] and a significant effect improving the immune system without affecting the intestinal microbiota in dairy calves [29]. However, no studies have been published to determine the effect of essential oils on CH_4_ intensity in commercial dairy cows associated with productive impact variables such as fat, protein, and milk yield and the quality of the pasture at different times of the year in grassland-based productive systems. Our work aimed to evaluate the effect of EO inclusion in concentrate feed as part of the diet of milking cows at a two-seasonal grassland-based farm on methane intensity and production performance, considering the nutritional quality of grasslands production in the dairy systems of southern Chile, in a particularly warmer spring–summer season.

## 2. Materials and Methods

### 2.1. Location and Climate Data

The study was conducted during spring–summer (September 2023–January 2024) at the commercial dairy farm Agrícola Folilco Ltd., located in the Folilco county in Río Bueno, Los Ríos Region, Chile (40°26′22.7″ S–72°38′43.3″ W). The study area has a temperate climate with an average annual rainfall of 1024.2 mm, distributed throughout the year, and the mean daily minimum and maximum temperatures range between 5.2 and 6.5 °C and between 16.5 and 16.9 °C, respectively. Agrícola Folilco Ltd. (Río Bueno, Los Ríos Region, Chile) uses a bi-seasonal dairy production model, with calving concentrated in two periods each year: spring and fall. The dairy farm operates primarily on pasture, with a maximum supplementation of 25% from concentrated feed or silage relative to pasture consumption. At the time that this study was performed, the herd had 449 cows on the dairy platform, representing a blend of genetic variants, including Red Friesian, Black Friesian, Jersey, and Montbeliarde. The crossbreeding used aimed at improving fertility and probably also the survival of the offspring at birth (Montbeliarde), as well as the fat yield (Jersey) or the protein yield (Red Friesian, Black Friesian) [25]. The average milk production reached 20.94 ± 6.38 kg/d (average ± standard deviation). The visual assessment and the farmer provided information on floral composition, which showed a dominance of perennial ryegrass (*Lolium perenne*; 70%), other grass (*Lolium multiflorum* and *Paspalum dilatatum*; 18%), white clover (*Trifolium repens*; 10%) and broad-leaf weeds (2%). Also, the soil was characterized as an Andisol soil type, Puerto Fonck series, Trumao [30].

**Figure 1:**
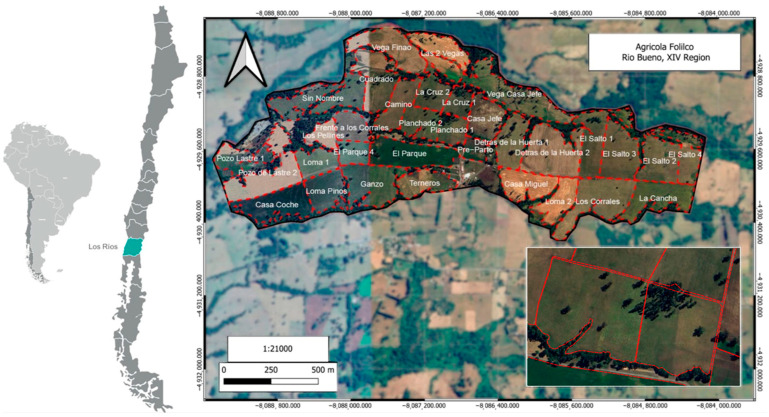
Location and layout of the paddocks Agrícola Folilco Ltd. (Río Bueno, Los Ríos Region, Chile). This commercial dairy farm is located at Río Bueno, Los Ríos Region (XIV), Chile. It operates a structured rotation system for herd movement across designated paddocks throughout the year. Farm limits are shown with inner paddocks in red dashed lines. Inset shows the paddocks, Planchado 1 and Planchado 2, in which the trial was performed. The map indicates UTC coordinates and WGS84 pseudomercator projection.

In southern Chile (Figure 1), the most widely used dairy production system (75%) is free grazing, based on the direct grazing of permanent and improved grasslands as the main feed [31,32,33]. Milk production in these systems is linked to available forage consumption and quality [34]. National research has shown that, during the spring, and under appropriate management, cows can achieve milk yields of between 20 and 24.5 kg per day from grazing alone [35]. Although, dairy production systems based on rotational paddocks for permanent year-round grazing are the lowest cost feed source [36], and these systems use accurate grazing management to ensure an adequate daily allocation of high-quality forage [26,36]. This makes it a laborious and time-consuming activity due to the need for regular pre- and post-grazing measurements and the continuous movement of the herd [12]. In Agrícola Folilco Ltd. (Río Bueno, Los Ríos Region, Chile), the area of 417 ha is divided into 32 paddocks (Figure 1) in which 27 grazing strips are used for daily forage allocation. The paddocks have approximately 9.78 ± 3.82 ha, and rotation is carried out with a frequency of 60 days in winter and 30 days in summer, with a significant drop in yield/ha during the summertime [33]. The inset highlights the two specific paddocks (Planchado 1 and Planchado 2) assigned to the experimental group during the trial period. These paddocks were representative of the forage (quality and availability) received by the whole herd during the same period.

### 2.2. Animals and Experimental Treatment

The dairy farming system at Agrícola Folilco Ltd. consists of 499 cows, classified into three categories: (i) general milking parlour (dairy cows; *n* = 449, 89.97%), (ii) dry cows (*n* = 45, 9.01%), and (iii) others (*n* = 5, 1.02%). Using a randomized block design, thirty-four mid–early lactation cows (97.2 ± 45.6 DIM, days in milk) were selected at the beginning of September, which had an overall average milk yield of 20.94 ± 6.38 kg/d (Table 1). Initial cow selection for the assay was performed based on a randomized sample from the total herd based on the management system available in the farm and considered parity, days in milk, and milk yield, leading to two selected groups with no significant differences in those parameters (Table 1). The pre-experimental group of 34 cows had an average milk yield of 22.3 ± 5.37 kg/d and an average parity of 3.42 ± 1.13. These animals underwent a two-week training period to adapt to the methane measurement system (Greenfeed^®^ (GF), C-LOCK, Rapid City, SD, USA) in which 30 cows were selected based on behaviour and management criteria. An additional two-week covariate period was conducted. And then a ten-week experimental period began in late September to early January.

Both groups received the same basal feed consisting of 16.44 kg/d of DMI composed by grassland, silage, molasses, and concentrate. Also, drinking water was always freely available, both in the pasture and in the milking parlour holding pen. Groups received 2.25 kg of concentrate feed (as offered), in which treated cows received concentrate supplemented with EO. Both feeds were manually administered in the milking parlour during each milking at 03:30 and 15:30 h throughout the treatment period. Consumption was monitored by visual observation by the staff. The concentrate was stored in the farm storage house at room temperature (20–25 °C). The EO-supplemented concentrate contained two commercially available mixtures: Valkalor^®^ (Eugenol, Geranyl acetate, and fruit aroma) and Vertan^®^ (containing Eugenol, Linalool, Geraniol, Thymol, and vanilla aroma). The final inclusion of EO was 0.35% (3.5 g/kg of concentrate), equivalent to 7.9 g of EO mixture per cow per day, based on the 2.25 kg of concentrate consumed. These mixtures were manufactured from mineral carriers (sodium sulphate and calcium carbonate) with EO obtained through chemical synthesis (Spain, England, Belgium). The commercial concentrate was made with fibrous by-products (450 g/kg), oats, soybean, and wheat (510 g/kg in total) and minerals and additives (40 g/kg). The complete diet composition during the step-up adaptation protocol is shown in Table 2.

Body condition was assessed using an individual record of the animals that was kept through measurements taken once per week (during the whole trial) after the morning milking. The visual estimation of body condition (BC) was made using the Elanco scale, which assigns CC values from 1 to 5, where 1 corresponds to a thin animal and 5 to a fat animal (Supplementary Table S1). Staff were trained using the DairyNz Guideline [37].

### 2.3. Routine Grasslands and Sampling

The grassland-based diet was provided on a rotational grazing basis, with the experimental paddock divided into 0.18 ha strips. Silage and molasses were distributed as TMR after milking at the paddocks assigned to the trial. Dry matter intake (DMI) per animal was estimated based on the quantity offered and the number of animals per group, according to farm records. The NDS professional software with CNCPS v6.55/v6.56 was used to calculate nutrient composition and total diet. Milking typically occurred between 03:30 and 05:30 h and between 15:30 and 17:30 h. Milk composition samples were also taken fortnightly (at weeks 0, 2, 4, 6, 8, and 10) from Wednesday afternoon to Thursday morning using milk sampling equipment, adapted from Afimilk system (https://www.afimilk.com). The samples of the individual fresh milk were collected in 10 mL flasks containing the conservative bronopol, without touching the inside of the sample container. Immediately after collection, the samples were placed in the insulated sample storage case at 4 °C to be transported to the laboratory within 12 h. These individual milk samples were analyzed using a MilkoScan FT7 RM (Foss Electric, Eden Prairie, MN 55344, USA) for protein, fat percentages, milk urea concentration, and somatic cell count (SSC). Milk solids and urea were analyzed using the infrared MilkoScan method (ISO 9622/IDF 141:2013) [38] and (ISO 8196-2/IDF 128-2:2009) [39]. SCC was performed using the fluoro-opto-electronic method (ISO 13366-2/IDF 148-2:2006) [40].

Blood samples were collected individually from the middle coccygeal vein puncture and stored in tubes without anticoagulant (Becton, Dickinson and Company, BD Vacutainer^®^ blood collection tubes, Franklin Lakes, NJ, USA). Sampling was performed in weeks 0, 2, 4, 6, and 8 of the experimental periods, with one sample per cow per collection day, resulting in a total of five samples per cow. Samples were stored at 4 °C and sent to a commercial hematology lab for analysis within 24 h for a complete metabolic profile under standard ISO 17025 [41]. We analyzed aspartate aminotransferase (AST), alkaline phosphatase (ALP), gamma glutamyl transpeptidase (GGT), non-esterified fatty acids (NEFA), total bilirubin, lactic dehydrogenase (LDH), total cholesterol, beta-hydroxybutyrate (BHB), alanine aminotransferase (ALT), albumin, total protein, and urea.

Forage samples were collected biweekly from the experimental paddocks, following a zig-zag protocol and avoiding trees, waterers, and edges. Samples (~500 g) were cut 7–9 cm above ground, stored at 4 °C, and analyzed within 12 h. Near-infrared spectroscopy (NIRS, FOSS DS2500, Ciudad de México, Mexico) was used to determine ash, fatty acids (C18:3, C18:2, C18:1, C18:0, C16:1), crude fat, starch, total sugars, lignin, ADF, NDF, amino acids, soluble protein, dry matter, and humidity. Crude protein was confirmed by nitrogen combustion [42] (Leco FP-528, St. Joseph, MI, USA), and metabolizable and net lactation energy were calculated using OARDC summative equations [43]).

### 2.4. Methane Emissions

Enteric methane (CH_4_) emissions were measured using a greenhouse gas (GHG) system from C-Lock Inc., which recorded individual animal emissions for the whole experimental period. The GHG system was placed in the waiting area of the dairy farm. GHGs were measured twice a day before the experimental group entered the milking parlour. The GF was programmed using C-Lock Inc. software SmartFeed™ to dispense a maximum of 18 rotations of a feed dispenser cup. Each rotation delivers approximately 55–60 g of concentrate, as bait with non EO-supplemented concentrate, with 10 s intervals between each rotation. An amount equivalent to 1.08 kg of concentrate was delivered during each visit. This bait program was used for all animals that were included in the trial.

### 2.5. Statistical Analysis

Individual cow milk yield was measured daily using Serlac DataFlow™ II software (SERLAC Software, Valdivieso 610, Llanquihue, Chile). The correction of milk production by the 4% fat content (4% FCM) during the trial period was performed by the following formula [44]:

(1) 4% Fat corrected Milk (kg)=0.4×kg milk+15×kg fat

The correction of milk production as Energy Corrected Milk (ECM) during the trial period was performed by the following formula [45]:

(2) $$ECM\;(\frac{kg}{day})=\frac{Milk\;yield\times ((Fat\;\%\times 0.383)+(Protein\;\%\times 0.212)+0.7832)}{3.1128}$$

Furthermore, gas data and agrometeorological information were acquired by C-LOCK GF^®^ data API (https://portal.c-lockinc.com/api/ accessed on 19 July 2025) and the Chilean agrometeorological service (www.agrometeorologia.cl), respectively. All statistical analyses and data visualization were handled under the R programming environment and R Studio (R Core Team, version 4.4.3, 2025; https://www.r-project.org/). Statistical significance was fixed at *p* < 0.05. Gas data quality assurance was performed by filtering the dataset, including only measurements equal to or more than 2.5 min. for animal maintenance on the Green Feed equipment. ANOVA was applied to compare the significance between the two groups. Values are given as mean ± SD.

## 3. Results

### 3.1. Herd Performance and Seasonal Forage Variation

The ingredient and nutrient composition (%DM basis) of the diet in the step-up adaptation protocol for both experimental groups and the mean intakes of dry matter intake (kg/d), crude protein (%) and neutral detergent acid fibre (%) were, respectively, 13.9 ± 3.6; 14.8 ± 0.2; and 30.2 ± 1.4, as shown in Table 2.

**Figure 2:**
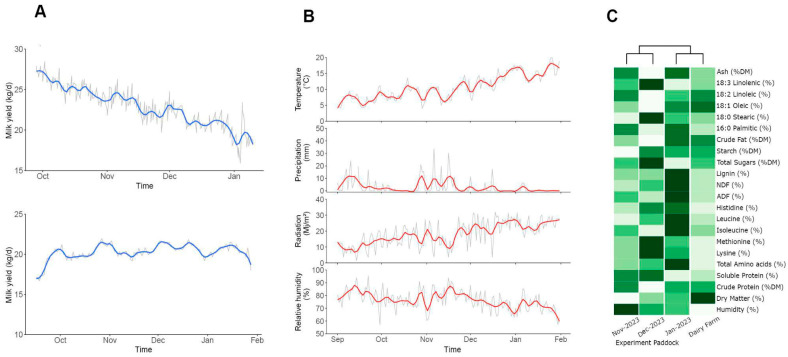
**Productive performance, environmental conditions, and grassland composition during the trial.** Mean values are shown as grey lines and colour solid lines represent the moving average of 3 consecutive values. (**A**) The experimental group (top) and the whole herd (bottom) lactation curve during the trial period. (**B**) Meteorological data from October to February: Temperature (°C), precipitation (mm), solar radiation (MJ/m^2^), humidity (%). (**C**) Nutritional indicators heat map of NIR-analyzed grassland including ash, fatty acids (18:3, 18:2, 18:1, 18:0, 16:1), crude fat, starch, total sugars, lignin, ADF, NDF, amino acids (histidine, leucine, isoleucine, methionine, lysine, total), soluble protein, dry matter, and humidity. Mean values are shown as colour scale and row data is in Supplementary Table S2.

Many dairy farms in the Southern Region of Chile favour using body condition score (BCS) over installing a weighing scale, as it is the case at Agrícola Folilco Ltd. Therefore, we use the BCS as a proxy for health and productivity of the experimental group as well as to monitor any changes in the cows during early lactation that account for a significant modification in the energy metabolism associated with the biweekly metabolic profile [37,46]. The BCS of the cows of both groups was 2 ± 0.25 (Supplementary Table S1). with an average milk yield (MY) of 20.94 ± 6.38 kg/d/cow. The lactation curve of the experimental group (treated and control cows) is shown in Figure 2A (upper panel), with a normal progression of days in milk for both groups. The bottom panel of Figure 2A shows that the milk production of the whole herd during the experimental period was stable, due to the permanent entry of cows in stages of early lactation to the dairy parlour. These data highlight that the trial was conducted in a commercial dairy farm with continuous annual production that never goes below 22 kg/d/cow. Milk protein and fat contents were 3.74 ± 0.2 and 4.8 ± 1.4%, respectively.

The meteorological data for the whole trial period is shown in Figure 2B from the Instituto de Investigaciones Agropecuarias (INIA) meteorological station of Rucatayo, Río Bueno, Los Lagos Region, Chile. Data were taken for solar radiation (W/m^2^), relative humidity (H°), precipitation (mm), and temperature (°C). Significant variations in radiation (18.01 ± 7.7), humidity (76.4 ± 7.8), and temperature (10.6 ± 3.8) were observed from the middle of November and were maintained until the end of the trial (February). These variations are related to changes in the nutritional paddock’s grassland composition (Figure 2C and Supplementary Table S2). The results showed an absolute increase in neutral digestible fibre and acid digestible fibre (NDF and ADF, 4.60% and 2.41%, respectively) and non-structural carbohydrates, such as lignin (1.48%). However, there was a decrease in soluble and crude protein (1.51% and 1.16%), total sugars (2.89%), and humidity (4.44%), representing a major shift in the main nutritional characteristics of grassland.

### 3.2. Performance and Methane Emissions

#### 3.2.1. Performance

**Figure 3:**
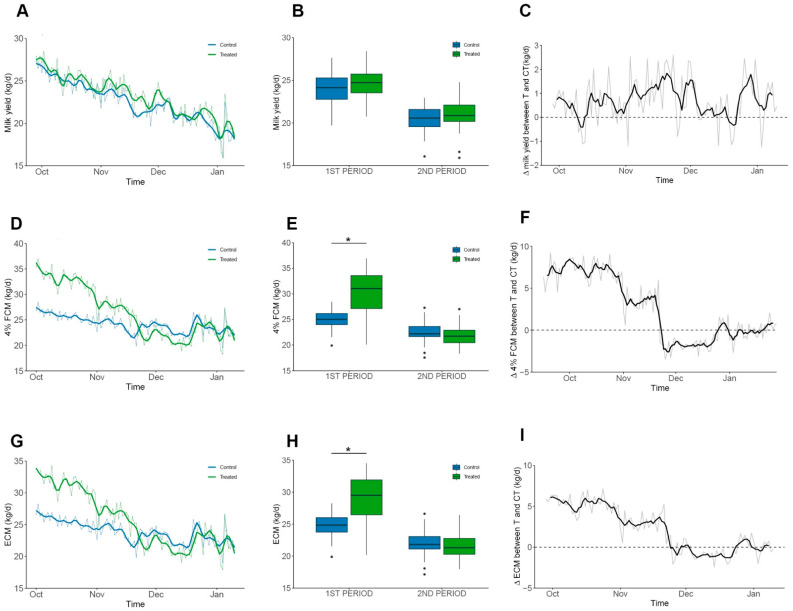
**Milk production performance.** Milk production parameters in the experimental period. Groups are shown in colours blue and green for Control and Treated, respectively. Mean values are shown as grey lines and colour solid lines represent the moving average of 3 consecutive values. Plots (**C**,**F**,**I**) represent the difference between daily average values. (**A**–**C**) Daily milk yield (kg/d). No significant differences were detected within periods and average difference was 0.6–1.0 kg/d without statistical significance. (**D**–**F**) 4% FCM (kg/d). Observed average difference was 2.4 kg/d with a significant difference in the first period (4.6 kg/d; *p* < 0.001). (**G**–**I**) ECM (kg/d). Observed average difference was 2.3 kg/d, with a significant difference during the first period (4.1 kg/d; *p* < 0.0013). Significant data is denoted by “*” symbol.

The assay was divided into two periods determined by weather changes observed in mid-November (Figure 2B): The first period had low temperature and high humidity, and the second period had high temperature and low humidity. At the beginning of the assay, no significant differences were observed over raw milk yield (22.68 ± 2.48 kg/d and 21.99 ± 2.31 kg/d, control and treated group, respectively). Throughout the experimental period, the treated group maintained the difference, producing an average of 0.7 kg/d more raw milk than the control group (Figure 3A–C). Although, this difference was not statistically significant in any period (*p* = 0.62). However, significant differences were observed in 4% fat-corrected milk (FCM) within the first period, reaching 23.9 ± 2.2 kg/d and 26.4 ± 5.3 kg/d for the control and treated groups, respectively (Figure 3C,D). Also, significant differences were observed when corrected to energy-corrected milk (ECM) for the same period, reaching 23.6 ± 2.2 kg/d versus 25.7 ± 4.7 kg/d for the control and treated group, respectively (Figure 3E,F; *p* < 0.001). Additionally, EO supplementation did not affect the SCC of milk (*p* = 0.8), although the treated group had numerically lower values compared to the control (0.82 ± 2.206 vs. 2.235 ± 4.438 × 10^3^ cells/mL; Supplementary Table S3).

#### 3.2.2. Herd Methane Production and Intensity

**Figure 4:**
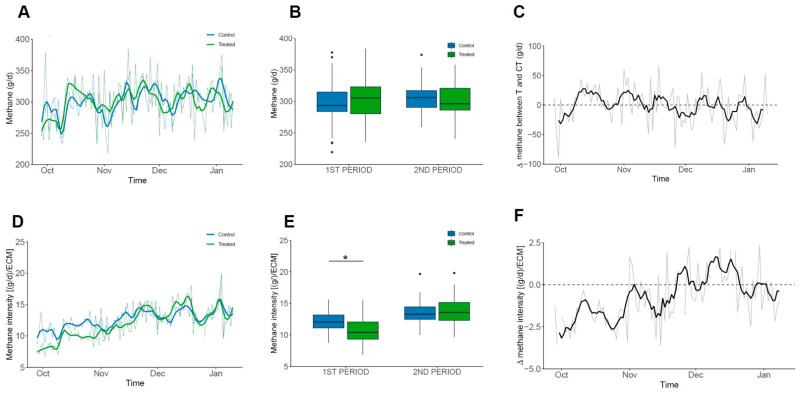
Methane Production and Intensity in Grazing Cows. Groups are shown in colours blue and green for Control and Treated, respectively. Mean values are shown as grey lines and colour solid lines represent the moving average of 3 consecutive values. Plots (**C**,**F**) represents the difference between daily average values. (**A**–**C**) Daily methane emissions (g/d). No significant differences were detected within periods. (**D**–**F**) Methane intensity [(g/d)/ECM]. Treated cows showed consistently lower values, with an overall average reduction of 0.7 and a significant difference during the first period (1.3; *p* < 0.001). Significant data is denoted by “*” symbol.

The grassland-based diet of dairy cows has highly desirable comparative advantages when compared to milk production with cows in confinement [47,48]. The most important of these are the cost of production, improved solids quality, and animal welfare [49]. Regarding methane emissions, the individual methane emission values recorded by the GF^®^ system in this trial, 301.14 ± 30.4 g/d/cow, are less than the previously published 361 g/d/cow [20,50]. The varied genetic background of the herd at Agrícola Folilco Ltd., combined with the variability of the paddocks producing pastures of differing quality, leads to individual differences in gross methane emissions (grey line in the background of Figure 4A,C,D,F and Supplementary Table S4). This variability has been consistently observed across all commercial pasture-based dairies that our group has measured. Although no significant differences were observed between the control and treated groups when comparing total daily methane production (Figure 4B), a decrease in methane intensity was observed during the first period of the trial (Figure 4E,F) due to the significant change in fat concentration of the treated cows which resulted in an increase of 2.55 and 2.12 kg/d when corrected for both 4% FCM and ECM.

The grassland-based diet of dairy cows offers clear advantages over confinement systems, including lower production costs, higher milk solids quality, and improved animal welfare. In this trial, individual methane emissions measured for the cows averaged 301 g/day per cow, which is lower than previously reported values for similar systems. Differences in genetic background within the herd, together with variability in pasture quality across paddocks, led to noticeable individual variation in methane emissions. Although total daily methane production did not differ significantly between the control and treated groups, a reduction in methane intensity was observed during the first period of the trial. This was associated with a significant increase in milk fat concentration in the treated cows, resulting in higher fat-corrected and energy-corrected milk yields of 2.55 and 2.12 kg/day, respectively.

### 3.3. Biochemical Profile

The cows’ biochemical profile during the trial is shown in Table 3. No major fluctuations among physiological parameters were observed throughout the whole assay, and no major differences were observed between experimental groups, indicating the good herd health status. Further, energy metabolism indicators, such as NEFA, decreased from the first to the second period in both groups (from 171.21 to 120.41 µmol/L in control cows and from 176.52 to 117.24 µmol/L in treated cows), and β-hydroxybutyrate concentrations increased slightly in the second period (from 0.63 to 0.76 mmol/L in cows in the control group and from 0.65 to 0.71 mmol/L in cows in the treated group).

## 4. Discussion

Chilean dairy farms typically rely on year-round grazing, allowing cows to remain outdoors most of the year. Grassland growth occurs from early spring to late autumn, challenging the use of anti-methanogenic additives in these systems [35,36]. In temperate regions between 35 and 42° S, permanent grasslands are common, including naturalized, improved, and cultivated types which can be observed at the Folilco farm [12,26,51]. Seasonal growth and local climate strongly influence yield, which averages 12 t DM/ha at Folilco, with peak growth during spring and early summer [12,35]. Grassland growth is mainly driven by moisture and radiation, with optimal conditions in spring, supporting milk production of 20–24.5 kg/d under good management [32,47,52,53,54].

Nutritional analysis of experimental paddocks by NIR, performed fortnightly, revealed stable percentages of linoleic, oleic, and palmitic acids, while differences between November and December coincided with the increase in milk fat during the first phase of the trial [Supplementary Table S2; Figure 2C]. This aligns with known patterns, where α-linolenic acid is the dominant FA in pastures (50–75% of total FA), and unsaturated FA concentrations decline as grasses transition from vegetative to reproductive stages [18,48,55,56,57,58,59,60,61]. Non-esterified fatty acids (NEFAs), triglycerides, and beta-hydroxybutyrate (BHB) are key indicators of metabolic status, particularly in early lactation [18,62,63]. The observed differences in BHB may reflect either EO supplementation enhancing rumen biohydrogenation and VFA flow, the genetic selection of efficient cows in energy and nitrogen metabolism, or both [24,64].

Supplementation with essential oils did not significantly alter total daily methane emissions, but reduced methane intensity during the first trial period, coinciding with higher milk fat, 4% FCM and ECM, in treated cows [65,66,67]. These results are consistent with temporary reductions in methane intensity reported in other studies [65], though some trials with similar EO doses observed no effect [19,67]. Despite limited effects on methane emissions, EO supplementation altered milk composition, likely through de novo fatty acid synthesis in the rumen, stimulated by EO and supported by pasture protein availability [10,68,69,70,71]. Odd- and branched-chain fatty acids (OBCFA) in milk further suggest microbial involvement, which is linked to host genetics and rumen microbiome composition [64,72,73].

To date, no comprehensive sequencing data on rumen microbial modifications under grazing conditions with EO supplementation in commercial herds exist. Future work should explore microbiome dynamics across genetic backgrounds, lactation stage, and EO treatment to understand microbial contributions to milk production and methane metabolism.

## 5. Conclusions

Essential oil supplementation in pasture-based dairy systems reduces methane intensity and can improve milk fat and protein yields under specific pasture quality conditions. These effects are closely linked to forage composition, microbial activity, and cow genetics. Routine nutritional analysis of grasslands (e.g., NIR) is crucial to optimize additive effects. Effective methane mitigation strategies should integrate pasture quality, microbial activity, and animal genetics to maximize both environmental and productive outcomes.

## Figures and Tables

**Table 1:** Classification and distribution of cows at the dairy farm start of the trial.

	Herd			Exp. Group	
Parameter	High Pens	Average Pens	Lower Pens	Treated	Control
Number of cows	217	449	232	15	15
Milk yield (kg/d)	25.13 ± 5.07	20.94 ± 6.38	16.88 ± 4.67	22.68 ± 2.4	21.99 ± 2.3
Days in milk	154.19 ± 121.74	183.46 ± 115.64	207.6 ± 104.68	124.3 ± 81.62	123.9 ± 82.7
Parity (N°)	3.55 ± 1.72	3.29 ± 1.85	3.01 ± 1.94	3.1 ± 0.9	3.6 ± 1.17

**Table 2:** Ingredient and nutrient composition (%DM basis) of the diet protocol for both experimental groups, considering the training period, covariate period, and experimental span.

Composition ^1^	Grassland	Silage	Molasses	Concentrate	Total
DMI ^2^ (kg/d)	6	6.08	0.4	3.99	16.44
CP (%)	21.38	12.54	8.49	18.55	
NEL(MJ/kg)	1.75	6.19	1.57	1.81	
NDF (%)	36.02	47.32	-	15.76	
Crude fat (%)	3.08	2.27	0.2	2.52	

^1^ DMI (Dry Matter Intake), CP (Crude Protein), NDF (Neutral Detergent Fiber), NEL (Net Energy for Lactation). Manufactured by IANSA S.A. Animal Nutritional Products, Quepe Plant, Chile. ^2^ Grassland DMI was provided by the farmer’s estimation, which was considered as 6 kg.

**Table 3:** Effect of essential oils supplementation on biochemical profile during the trial (Mean ± SD).

Parameter	Unit	* (CT) 1st Period	* (T) 1st Period	(CT) 2nd Period	(T) 2nd Period	Reference Value
AST	U/L	101.7 ± 25.77	95.37 ± 21.59	78.36 ± 19.17	78.06 ± 16.75	2–110
ALP	U/L	63.18 ± 27.65	71.01 ± 42.53	74.76 ± 57.52	70.66 ± 32.87	0–196
GGT	U/L	24.79 ± 5.53	22.33 ± 5.33	23.95 ± 4.9	23.36 ± 4.53	3–39
NEFA	µmol/L	171.21 ± 38.38	176.52 ± 54.25	120.41 ± 52.03	117.24 ± 60.02	100–600
LDH	U/L	1247.04 ± 164.72	1229.43 ± 212	1136.83 ± 163.79	1135.66 ± 147.36	692–2840
Total cholesterol	mmol/L	4.53 ± 1.03	4.72 ± 1.03	4.67 ± 1.02	4.54 ± 1.1	2.7–5.3
ALT	U/L	31.2 ± 6.48	32.78 ± 7.86	30.68 ± 5.72	31.4 ± 7.03	<55
Albumin	g/L	35.35 ± 3.05	35.89 ± 3.3	36.16 ± 3.12	36.7 ± 2.78	29–41
Total protein	g/L	88.47 ± 7.11	87.23 ± 5.94	83.17 ± 6.27	83.25 ± 7.19	66–90
Triglycerides	mmol/L	0.47 ± 0.03	0.48 ± 0.03	0.42 ± 0.03	0.43 ± 0.02	0.1–0.3
Urea	mmol/L	4.16 ± 1.12	4.3 ± 1	4.68 ± 0.82	4.91 ± 1.03	2.6–7.0
Total bilirubin	µmol/L	2.99 ± 1.77	3.28 ± 1.85	5.01 ± 0.68	5.06 ± 0.68	0.2–7.8
β-hydroxybutyrate	mmol/L	0.63 ± 0.15	0.65 ± 0.17	0.76 ± 0.14	0.71 ± 0.14	0.1–0.6

* CT: Control group; T: Treated group.

## Data Availability

The data presented in this study are available from the corresponding author upon request. Sensitive data, which are protected by the non-disclosure agreement between the University and the producer, may eventually be shared with consent.

## Supplementary Materials

The following supporting information can be downloaded at https://www.mdpi.com/article/10.3390/ani15243549/s1, Table S1: Animals body condition; Table S2: Nutritional Near Infrared (NIR) of the pastures; Table S3: Milk quality (Mean ± SD); Table S4: Variance Analysis of Effect of essential oil (EO); Table S5: Methane and carbon dioxide individual recordings. GF—Raw data.

## Abbreviations

The following abbreviations are used in this manuscript:

EO	Essential Oils
BCS	Body Condition Score
BHB	Beta-Hydroxybutyrate
CP	Crude Protein
CH_4_	Methane
DM	Dry Matter
DMI	Dry Matter Intake
ECM	Energy-Corrected Milk
FCM	Fat-Corrected Milk (4%)
GHG	Greenhouse Gases
ID	Identifier
ISO	International Organization for Standardization
LDH	Lactate dehydrogenase
MY	Milk Yield
NE	Net Energy
NEFA	Non-Esterified Fatty Acids
NDF	Neutral Detergent Fiber
NIR	Near-Infrared Spectroscopy
SCC	Somatic Cell Count
SD	Standard Deviation
TMR	Total Mixed Ration
VFA	Volatile Fatty Acids
