# Supplementary material for: Use of Essential Oils in the Diet of Lactating Cows Enhances Productivity and Reduces Methane in Free-Grazing Commercial Dairy Farms
Source: Animals (Basel). 2025 Dec 10;15(24):3549. doi: 10.3390/ani15243549 (PMC12729745; doi:10.3390/ani15243549)
Supplement: Supplementary file 1 [file animals-15-03549-s001.zip › animals-3798480-supplementary.pdf]

SUPPLEMENTARY MATERIAL

Supplementary Table S1. Animal body condition

Group	Period	BCS * (Media ± SD)
Control	1st Period	2.5 ± 0.25
	2nd Period	3 ± 0.5
Treated	1st Period	2.5 ± 0.25
	2nd Period	3 ± 0.5

* Body Condition Score (BCS).

Supplementary Table S2. Nutritional indicators of the dairy farm pastures analyzed by Near Infrared (NIR) of the pastures used for grazing the cows in the trial.

Parameter	November	December
Ash (%DM)	8.69	7.85
18:3 Linolenic (%)	60.625	61.34
18:2 Linoleic (%)	14.74	14.235
18:1 Oleic (%)	3.78	3.39
18:0 Stearic (%)	2.93	3.175
16:0 Palmitic (%)	16.035	15.83
Crude fat (%DM)	4.41	4.19
Almidón (%MS)	2.68	3.42
Total sugars (%DM)	19.27	21.265
Lignin (%)	2.835	3.435
NDF (%)	40.56	42.56
ADF (%)	23.95	24.3
Histidine (%)	1.135	1.16
Leucine (%)	4.76	4.82
Isoleucine (%)	2.345	2.31
Methionine (%)	1.435	1.505
Lysine (%)	3.63	3.785
Total amino acids (%)	60.52	60.49
Soluble protein (%)	31.855	32.725
Crude protein (%)	19.835	17.345
Dry matter (%)	15.45	18.005
Humidity (%)	84.55	81.995

Supplementary Table S3. Milk quality (Mean ± SD)

Condition	Period	Fat (%)	Protein (%)	SCC * (cells/mL) × 103	Urea (mg/L)
Control	1st period	4.27 ± 1.26	3.80 ± 0.21	108	293.79 ± 70.30
Treated	1st period	5.43 ± 1.61	3.71 ± 0 .26	81	280.88 ± 65.39
Control	2nd period	5.06 ± 1.54	3.67 ± 0.34	114	339.45 ± 88.28
Treated	2nd period	4.80 ± 1.02	3.64 ± 0.25	81	317.44 ± 101.70

* Somatic Cell Count (SCC) is expressed in quartiles.

Supplementary Table S4. Effect of essential oil (EO) through Greenfeed® (GF) units over 14 weeks on enteric gas emissions from dairy cows on a commercial farm.

			**P value**		
**Item**	**Control (CT)**	**Treated (T)**	[1] **T1**	[2] **P2**	**T1 × P2**
CH_4, (g/d)	301.20 ± 30.28	301.07 ± 31.19	0.976	0.484	0.318
CH_4 yield, (g/kg milk yield)	13.58 ± 2.36	13.13 ± 2.27	0.117	**< 0.001**	0.535
CH_4 intensity, (g/kg ECM)	12.64 ± 1.82	11.93 ± 2.70	**< 0.05**	**< 0.001**	**< 0.01**
CO_2, (g/d)	10044 ± 600.96	10011 ± 616.40	0.688	**< 0.01**	0.490
CO_2, (g/kg leche)	451.31 ± 57.19	435.03 ± 53.73	**< 0.05**	**< 0.001**	0.839
CO_2, (g/kg ECM)	420.90 ± 42.31	394.63 ± 72.08	**< 0.001**	**< 0.001**	**< 0.001**

[1] T1 = Experimental conditions [2] P2 = Period.

Supplementary Table S5. Methane and carbon dioxide individual recordings. GF - Raw data.

https://1drv.ms/x/c/5798da0b1b052a0b/EealYaPDMMJIlVR713EW8aIB_1gyzuuU8JiY7LxwhHfxLQ?e=guPGiI
